# Impact of Visual Biofeedback of Trunk Sway Smoothness on Motor Learning during Unipedal Stance

**DOI:** 10.3390/s20092585

**Published:** 2020-05-01

**Authors:** Carlos Cruz-Montecinos, Antonio Cuesta-Vargas, Cristian Muñoz, Dante Flores, Joseph Ellsworth, Carlos De la Fuente, Joaquín Calatayud, Gonzalo Rivera-Lillo, Verónica Soto-Arellano, Claudio Tapia, Xavier García-Massó

**Affiliations:** 1 Clinical Biomechanics Laboratory, Department of Physical Therapy, University of Chile, 8380453 Santiago, Chile; carloscruz@uchile.cl (C.C.-M.); cristianrings@gmail.com (C.M.); dantemfh@gmail.com (D.F.); joseph.ellsro12@gmail.com (J.E.); gbrivera@uchile.cl (G.R.-L.); 2 Biomechanics and Kinesiology Laboratory, Hospital San José, 8380419 Santiago, Chile; 3 Department of Physiotherapy, Faculty of Heath Sciences, University of Malaga, 29071 Málaga, Spain; acuesta@uma.es; 4 Institute of Biomedical Research in Malaga (IBIMA), 29010 Málaga, Spain; 5 School of Clinical Science, Faculty of Health Science, Queensland University Technology, Brisbane, QLD 4000, Australia; 6 Carrera de Kinesiología, Departamento de Cs. de la Salud, Facultad de Medicina, Pontificia Universidad Católica, 7820436 Santiago, Chile; delafuentte@gmail.com; 7 Laboratorio LIBFE, Escuela de Kinesiología, Universidad de los Andes, 7620086 Santiago, Chile; 8 Centro de Salud Deportiva, Clínica Santa María, 7520378 Santiago, Chile; 9 Exercise Intervention for Health Research Group (EXINH-RG), Department of Physiotherapy, University of Valencia, 46010 Valencia, Spain; joaquin.calatayud@uv.es; 10 Neuroscience Department, University of Chile, 8380453 Santiago, Chile; 11 Research and Development Unit, Clínica Los Coihues, 9190025 Santiago, Chile; 12 Hospital Roberto del Río, 8380418 Santiago, Chile; vsotoarellano@gmail.com; 13 Universidad Tecnológica de Chile INACAP, Escuela Salud, 8340536 Santiago, Chile; 14 Human Movement Analysis Group (HuMAG), University of Valencia, 46022 Valencia, Spain; xavier.garcia@uv.es

**Keywords:** motor learning, biofeedback, postural balance, inertial measurement unit

## Abstract

The assessment of trunk sway smoothness using an accelerometer sensor embedded in a smartphone could be a biomarker for tracking motor learning. This study aimed to determine the reliability of trunk sway smoothness and the effect of visual biofeedback of sway smoothness on motor learning in healthy people during unipedal stance training using an iPhone 5 measurement system. In the first experiment, trunk sway smoothness in the reliability group (*n* = 11) was assessed on two days, separated by one week. In the second, the biofeedback group (*n* = 12) and no-biofeedback group (*n* = 12) were compared during 7 days of unipedal stance test training and one more day of retention (without biofeedback). The intraclass correlation coefficient score 0.98 (0.93–0.99) showed that this method has excellent test–retest reliability. Based on the power law of practice, the biofeedback group showed greater improvement during training days (*p* = 0.003). Two-way mixed analysis of variance indicates a significant difference between groups (*p* < 0.001) and between days (*p* < 0.001), as well as significant interaction (*p* < 0.001). Post hoc analysis shows better performance in the biofeedback group from training days 2 and 7, as well as on the retention day (*p* < 0.001). Motor learning objectification through visual biofeedback of trunk sway smoothness enhances postural control learning and is useful and reliable for assessing motor learning.

## 1. Introduction

Motor learning is a set of internal processes associated with practice and experience that lead to relatively permanent changes in the capability to perform a skilled behaviour. Depending on the task, this learning can be evaluated through the number of errors or successful/failed attempts during task execution and motor skills, such as postural control performance [1]. Postural control, defined as “the act of maintaining, achieving or restoring a state of balance during any posture or activity” [2], is essential for standing and walking independently in order to carry out many daily routine and sports activities [2,3]; this control is affected in older people and in several neurological and musculoskeletal diseases [4,5,6,7,8,9].

Postural control training is important to prevent falls, and control must be regained after neurological and musculoskeletal disorders [10,11,12,13]. Unipedal stance is a common exercise to assess and improve postural control [5,13,14,15,16,17,18]. Unipedal stance requires different configurations of the lower limbs and trunk, as well as inherent feedback from mechanoreceptors in the skin, muscle spindle, Golgi tendon organ and joint capsule [10,19,20,21,22,23]. To progress in postural control exercises, adequate integration of sensory and motor inputs is necessary in order to learn correctly [23]. Augmented feedback, understood as sensory information additional to inherent feedback, is a strategy used to reduce task errors, which is based on auditory, tactile or visual reinforcement stimuli [24,25].

A source of visual biofeedback may improve human balance, being a common tool employed in the training of postural control [26,27,28]; it appears to be effective for improving balance control among older adults [29], and exercise adherence through gamification [30]. In addition, it has been reported that visual biofeedback based on centre of mass (COM) displacement reflects the maximal capabilities of the balance control system and generates greater effort than biofeedback based on the centre of pressure (COP) [31]. To assess postural control, accelerometry of COM has been proposed as an economical and accessible tool that evaluates postural sway and balance improvement by biofeedback [5,6,32,33,34,35,36]. The accelerometer sensors embedded in smartphones have been proposed to have the potential to be a convenient, easy-to-use and valid tool for assessing postural control in normal and pathological populations [37,38,39,40,41]. In addition, smartphone device-based healthcare has appeared as a revolutionary approach to telerehabilitation practice [42].

Postural sway assessment reflects how the nervous system controls the complex sensorimotor task of maintaining unipedal–bipedal equilibrium [19,20,22]. The rate of change of acceleration, or ‘jerk’, has been used to assess the smoothness of postural control, where a higher value indicates less smoothness, reflecting the corrections made by the nervous system to control sway and maintain stance [43,44,45]. Postural sway jerk is a good marker of deterioration in Parkinson’s disease and multiple sclerosis [43,44,45,46] and can decrease after postural training in healthy young adults and patients with chronic spinal cord injuries [47,48]. However, there is little information about whether this postural control metric could be useful for detecting changes in the performance of balance tasks between days. This is relevant as smoothness of postural control based on jerk assessment using an accelerometer sensor embedded in a smartphone could be a biomarker for tracking motor learning in a clinical setting or during sports conditioning. This study aimed to determine the reliability of trunk sway smoothness and the effect of visual biofeedback of sway smoothness on motor learning in healthy people during unipedal stance training using an iPhone 5 measurement system. We hypothesized that trunk sway smoothness is reliable for assessing motor learning between days, and that visual feedback based on trunk sway smoothness enhances postural control learning.

## 2. Methods

### 2.1. Subjects

This study was approved by the local ethics committee and conducted in agreement with the Declaration of Helsinki. All participants were informed about the purpose and procedures of the project and gave their written informed consent to take part in the study. Exclusion criteria: signs or symptoms of injury or symptomatic arthritis to the trunk, lower back or lower limb within the past 3 months; history of musculoskeletal surgery in the lower limb or spine; scoliosis; history of acute or chronic musculoskeletal disorders; cardiac and/or respiratory pathology and neurological disease.

*Experiment 1 (reliability).* Eleven young men (23.6 ± 1.5 years, 1.74 ± 0.03 m and 73.2 ± 3.2 kg) were assessed on two days (three trials per day), separated by one week to determine the test–retest reliability of sway smoothness measurement without visual feedback.

*Experiment 2 (performance and motor learning comparison).* For the biofeedback group, 12 young men (25.7 ± 4.0 years, 1.77 ± 0.03 m, and 75.6 ± 4.2 kg) performed a training protocol consisting of seven sessions of training with 10 trials per day with visual feedback and one additional session with 10 trials without feedback (retention day). For the no-biofeedback group, 12 young men (26.9 ± 5.0 years old, 1.77 ± 0.07 m, and 75.6 ± 6.7 kg) performed a training protocol consisting of eight sessions with 10 trials per day without visual biofeedback. Both groups rested for one day between sessions. There was no overlap between the subjects of Experiments 1 and 2.

### 2.2. Procedures

**Figure 1:**
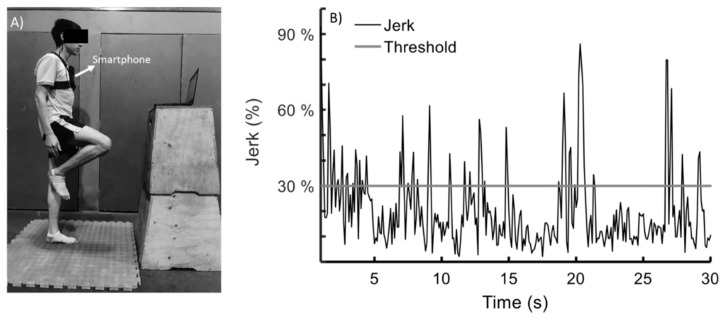
(**A**) Unipedal stance test. (**B**) The visual biofeedback signal was given at all times by a monitor (located 1 m away). The black signal represents the magnitude of the jerk vector of the centre of mass. The training threshold of 30% of the maximal jerk is represented with a grey horizontal line. Error was defined as time outside the training threshold during 30 s of unipedal stance.

For Experiments 1 and 2, all tests were conducted between 9 a.m. and noon. The participants maintained a unipedal stance over an unstable surface (density 60 kg/m^3^) with their dominant leg (Figure 1A). For the unipedal stance test, the participants were instructed to stand on one leg with a straight knee by lifting their opposite foot for 30 s, with their arms at their side and their eyes open. If the participant put both feet on the ground, the test was considered invalid and he was invited to repeat the test. COM acceleration was estimated with the triaxial accelerometer of an iPhone 5 measurement system (Apple Inc., Cupertino, CA, USA). The smartphone was fixed in a vertical position at the level of the sternum with a custom fixation (Figure 1A). Based on a recent systematic review, this location provides sufficient validity and intra-day reliability for kinematic evaluations of COM during dynamic balance tests [49]. iPod and iPhone accelerometers have been used in several studies which have shown that they are a valid tool to evaluate postural control [38,39,50,51].

For Experiment 2, visual feedback of sway smoothness was given by the magnitude of the jerk vector of the COM through an interface designed with MATLAB 2015 software (MathWorks Inc., Natick, MA, USA). For the visual feedback, subjects stood 1 m from the 14-inch laptop screen, with maximal brightness and a visual angle of ~45°. For the reliability group (experiment 1) and no-biofeedback group (Experiment 2), the instruction to the subject was to keep his vision focused on a 3 cm × 3 cm mark that was placed on the screen of the same laptop used for the biofeedback group.

### 2.3. Data Analysis

In assessing trunk sway smoothness, global trunk smoothness (along the three dimensions *x*, *y*, *z*) was taken into account as opposed to measuring only along a given axis [52,53]. For this, jerk was obtained instantaneously as the Euclidean distance from three orthogonal discrete accelerometry signals acquired from the smartphone ($G_{j}$), where j represents the inertial axis of the accelerometer, i.e., anteroposterior, mediolateral and superiorinferior axes relative to the COM of the accelerometer; nT represents periodic sampling; i represents the number of samples; and := means jerk update while acquisition is running.

(1) $$Jerk\left[nT\right]\;:=Fs^{-1}\sqrt{\overset{3}{\underset{j=1}{\sum }}{\left(G_{j}{\left[nT\right]}_{i}-G_{j}{\left[nT\right]}_{i-1}\right)}^{2}}$$

Data were transferred to a laptop using the MATLAB Mobile^TM^ application (MATLAB Support Package for Apple iOS Sensors). The smartphone sampling rate was 10 Hz [50]. It is worth mentioning that a previous study found COM oscillations ranging between 2.5 and 4.5 Hz during one-leg balance training [22]. For each participant, the training threshold was defined as 30% of the maximum jerk generated during three unipedal tests lasting 30 s. This threshold was selected based on a previous report with a similar methodology [53]. Error was defined as time outside the training threshold during 30 s of unipedal stance (Figure 1B).

For experiment 2, the performance evolution adjustment during training days (7 days, 70 trials) was carried out by means of the power law of practice [54,55,56], based on the formula below.

(2) $$Power\;Law:T_{N}=T_{A}+b\times N^{-a}$$

Here, $T_{N}$ is the error in the Nth trial; the error data between days were standardized by the normalized error for each subject considering the first trial on the first day. $T_{A}$ is the asymptotic lower bound of the error to perform the test; N is the trial count-number that was performed to complete a specific task; b is that part of the error in performing the test that can be eliminated through practice (how much the error changes in the correct execution of the test with respect to the first trial); and exponent a is a measure of the rate at which trial error decreases (improvement). It is computed as the positive value of the average rate of change from successive trials (or the ratio of the amount of change over the interval of all trials). To determine significant adjustment between the power law and the normalized error curve, the coefficient of determination (R^2^) of the non-linear model was calculated for each subject. In addition, the fit generated for the non-linear model was used to compare the progression of practice between groups.

### 2.4. Statistical Analysis

An a priori power analysis conducted using GPower (3.1.9.2 version) software (Heinrich-Heine-Universität Düsseldorf, Düsseldorf, Germany) showed that 12 subjects per group (i.e., biofeedback and no-biofeedback groups) for this design were sufficient to obtain a statistical power of 0.80 at a large effect size (Cohen’s *d* = 1.19) [57], with an α = 0.05.

*Experiment 1. Reliability.* Intraclass correlation coefficient (ICC), standard error of measurement (SEM), minimal detectable change (MDC) and a Bland–Altman plot were used to calculate the test–retest reliability of jerk measurement in the reliability group (expressed as time outside the error threshold). ICC values were interpreted according to frequently quoted guidelines: poor < 0.4, 0.4 ≤ fair < 0.6, 0.6 ≤ good < 0.75, and 0.75 ≤ excellent ≤ 1. All statistical analysis was performed in SPSS version 22.0 (IBM Corporation, Armonk, NY, USA).

*Experiment 2. Performance comparison.* The non-linear fit of the power law across 7 days of training (i.e., 70 trials) for each subject was used to compare the performance between groups. For this, time-series statistical analysis was applied using the MATLAB-based spm1d package for n-dimensional statistical parametric mapping (SPM) [58]. Group comparisons were then carried out using a two-tailed independent samples *t*-test. The fit model was considered significantly different if any values of SPM over performance evolution exceeded the critical threshold (α = 0.05). To compare the rate of performance (exponent) of the power law and R^2^ of the non-linear model between groups, an independent samples *t*-test was applied.

*Motor learning comparison.* The average normalized error across 8 days (performance days and the retention day) was used to compare the motor learning between groups. The Shapiro–Wilk test was used to confirm the normality of the data. For differences in motor learning between days, 2 (groups) × 8 (days) two-way mixed analysis of variance, with the group (biofeedback and no-biofeedback) as the between-subject factor and days as the within-subject factor, was considered for the statistical analysis. Greenhouse–Geisser correction was used if the assumption of sphericity, as checked by Mauchly’s test, was violated. If a significant interaction was found between factors, the simple mean effect (one-way repeated analysis of variance across times for each group) and simple pairwise comparisons (unpaired *t*-test between the two groups) were applied. Post hoc tests with Bonferroni correction were applied for all comparisons.

Statistical significance was established at *p* < 0.05. To determine the effect sizes, the partial eta squared ($n_{p}^{2}$ ≥ 0.01, $n_{p}^{2}$ ≥ 0.06, $n_{p}^{2}$ ≥ 0.14) and Cohen’s d (*d* ≥ 0.2, *d* ≥ 0.5, *d* ≥ 0.8) were calculated to indicate small, moderate or large effects, respectively.

## 3. Results

### 3.1. Experiment 1: Reliability of Jerk Measurement

**Figure 2:**
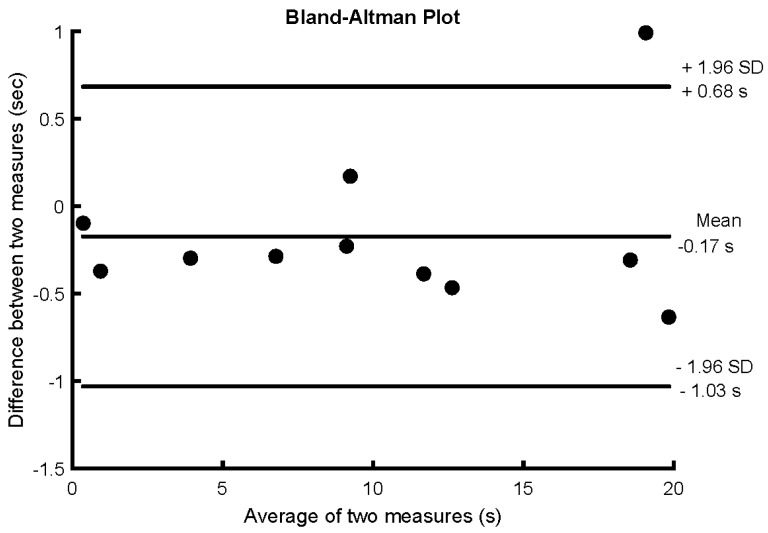
Bland–Altman plot of the reliability group for the difference in time outside the training threshold (i.e., 30% of maximal jerk) during 30 s of unipedal stance between two days (1 per week for 2 weeks) without biofeedback.

In the reliability group, no difference in balance performance was detected between days (10.10 ± 7.00 s vs. 10.27 ± 6.90 s; *p* = 0.218). The results had excellent test–retest reliability, with an ICC score of 0.98 (0.93–0.99), SEM of 0.30 s, MDC of 0.84 s equivalent to 2.8% of 30 s of balance registered, and the Bland–Altman plot showed a mean difference of 0.17 s (Figure 2).

### 3.2. Experiment 2: Performance and Motor Learning

**Figure 3:**
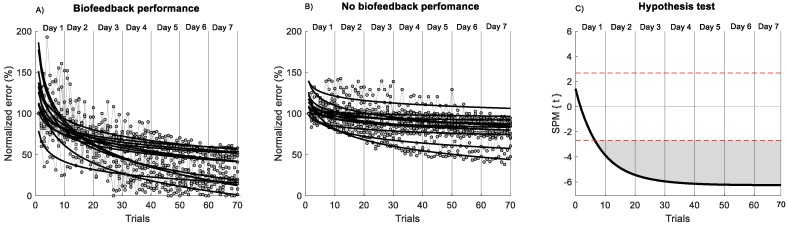
Non-linear model of power law across 7 days of training. (**A**) Biofeedback group. (**B**) no-biofeedback group. The black line indicates the non-linear model of the power law. The grey circle indicates the performance of each trial. (**C**) Hypothesis *t*-test (*t*) comparison of the non-linear fit of the power law between groups using statistical parametric mapping (SPM) analysis. The horizontal red dashed line indicates *p* = 0.05 level. Grey zones indicate regions with statistically significant differences (*p* < 0.001).

The SPM analysis applied to the non-linear fit of the power law across 7 days of practice (Figure 3) showed significantly better performance for the biofeedback group in comparison to the no-biofeedback group after the 10 trials of the first day (*p* < 0.001). In addition, a significant difference in the rate of improvement (exponent a) was observed across trials between groups (*p* = 0.003, *d* = 1.35) (Figure 3). Based on the normalized error curve, the rate of improvement for the biofeedback group was 0.64 ± 0.30% per trial; for the no-biofeedback group, it was 0.31 ± 0.20% per trial. The power law model provided a significant fit for all participants for both groups. A significant difference in R^2^ value was found between groups (*p* = 0.013, d = 1.11). The biofeedback group showed an R^2^ of 0.66 ± 0.10; for the no-biofeedback group, R^2^ was 0.48 ± 0.20. These results indicate that the performance of the biofeedback group was better than that of the no-biofeedback group and showed a better adjustment to non-linear power law fit. 

**Figure 4:**
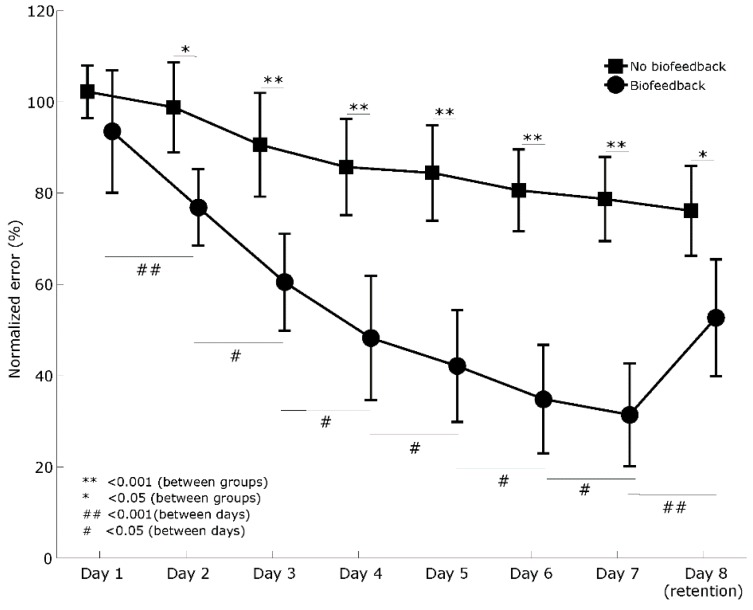
Comparison of the motor learning curves between groups and between days. Error bars indicate a 95% confidence interval.

Comparing the normalized error between days, two-way mixed analysis of variance indicated a significant main effect of group (F_(1, 22)_ = 30.3, *p* < 0.001, $n_{p}^{2}=$ 0.58) and a significant main effect of days (F_(2, 41)_ = 55.3, *p* < 0.001, $n_{p}^{2}$ = 0.72), as well as significant interaction between days and groups (F_(2, 88)_ = 11.2, *p* < 0.001,$\;n_{p}^{2}=$ 0.337). The simple mean effect of the biofeedback group (F_(2, 17)_ = 36.5, *p* < 0.001, $n_{p}^{2}$ = 0.77) and no-biofeedback group (F_(2, 24)_ = 22.9, *p* < 0.001, $n_{p}^{2}$ = 0.68) showed significant differences between days. However, only the biofeedback group showed significant differences between all consecutive days (Figure 4, Supplementary Table).

Comparing the first day of training with all days in the biofeedback group, the post hoc analysis revealed a significant difference between all training days and the retention day (i.e., day 8) (*p* < 0.05) (Supplementary Table).

Comparing the first day of training with all days in the no-biofeedback group, the post hoc analysis only revealed a significant difference between the last day of training (i.e., day 7) and the retention day (*p* < 0.001) (Supplementary Table).

Finally, the simple pairwise comparisons showed a significant difference between groups from training days 2 and 7, as well as the retention day (i.e., day 8) (Figure 4, Supplementary Table).

Overall, these results indicate that both groups showed a significant improvement over time; however, the performance and retention of the biofeedback group were better.

## 4. Discussion

In this study, we confirmed the hypothesis that trunk sway smoothness is reliable for assessing motor learning between days and that visual feedback based on trunk sway smoothness enhances postural control learning. Despite both groups showing an increment in performance over time, we found that the performance and learning of the biofeedback group differed substantially from those of the no-biofeedback group between days. To our knowledge, this is the first report that has used smoothness of postural balance based on jerk as biofeedback and a biomarker of motor learning.

An important step towards understanding the effects of visual feedback of trunk sway smoothness on postural control is to determine the reliability of the measurement methodology. The method proposed in this study presents excellent intra-rater reliability between days. In addition, the change of error between days in the biofeedback group was superior to the MCD of 2.8% for all participants. These results indicate that visual biofeedback of trunk sway smoothness may be used as a biomarker parameter of motor learning during postural training.

There is little knowledge about the motor learning curve during postural control training between days [22,53,59]. In this study, based on a power law fit model, both groups showed a decrement of error across the trial; however, the rate of the decrement was higher for the biofeedback group. In addition, we found that two days of unipedal training with visual biofeedback of trunk sway smoothness may sufficiently decrease the normalized error. In contrast, unipedal training without biofeedback decreases the normalized error after 7 days of unipedal balance training (Figure 3). These results indicate that the visual biofeedback used in the present study enhances performance and the motor learning process, giving additional information on COM smoothness, which is useful for increasing performance during unipedal stance. In healthy young subjects, visual feedback has been reported to reduce the amount of COP displacement and the sway velocity [25,31]. However, visual feedback may have decreased the proprioceptive contribution to the control of postural sway, increasing the dependence on visual input to guide the sway during postural tasks, affecting retention of the task [60]. A previous study in adolescents reported that visual feedback of the COP during a bipedal standing position on a seesaw may not be enough to appreciate changes in comparison to the control group [61]. This means that the presence of visual feedback does not guarantee improved motor performance during postural tasks, and/or that the number of trials may have an influence on the motor learning process (70 vs. 12 trials) [61]. Despite this, in our study, visual biofeedback of trunk sway smoothness generated a positive effect on motor learning (i.e., performance between days) and task retention (i.e., last day without feedback) in comparison to the control group. These results indicate that the additional visual information of trunk smoothness to reinforce motor learning may be a crucial tool for ruling out postural errors during trunk sway. This ability to rule out error during the task may be used as a biomarker of better integration of sensory and motor inputs, reflecting how the nervous system controls the complex sensorimotor task of maintaining postural balance [20,22,31].

It was previously reported that visual biofeedback based on COM displacement may better reflect the maximal capabilities of the balance control system and generate greater effort than that based on the COP [31]. Whether trunk sway smoothness could be used to track and improve balance performance during sports conditioning or in different populations with neurological musculoskeletal disorders needs to be confirmed. Furthermore, the learning effects of biofeedback of trunk sway smoothness with other modality task errors (e.g., auditory and haptic biofeedback) [25,62], as well as the effects of terminal versus concurrent biofeedback, need to be compared in future studies [61].

Finally, little is known about the dose–response relationship in postural control training. Monitorization of motor learning curves may help determine the dose–response relationship during postural control training and the difficulty of the task [3,63], helping in the design of individualized programmes and prescription of adequate training frequency (e.g. number of days of training). Importantly, the applied method can be used in a clinical setting at little cost in order to regain postural control after different neurological and musculoskeletal disorders.

The present study has several limitations. First, we used a threshold of 30% for the maximal jerk test without visual feedback based on a previous report with a similar methodology [53]. However, other methods of data dispersion (i.e., two standard deviations) during testing need to be demonstrated in future studies. It would be interesting to determine a methodology for selecting individual or representative limits of the error threshold. Second, after training days, we demonstrated only the retention of motor learning. However, the transfer of motor skills acquired during the training days was not assessed. It is therefore not possible to know the impact of decreased trunk sway smoothness on other static and dynamic balance tasks. Third, this study focused only on men, so future research should consider comparative studies between genders, particularly regarding gender and postural control [64]. Fourth, this study recorded only trunk sway in order to describe balance. Therefore, it was not possible to elucidate the motor strategies used between lower limbs and the trunk to control the sway balance across days of training. Finally, this study used an accelerometer sensor embedded in a smartphone to record trunk acceleration with a sample rate of 10 Hz; a more precise measurement system with a higher sample rate could have detected more subtle changes among trials. Despite that, in our study, we observed that the iPhone 5 measurement system was sufficient to find significant differences across days of training and between groups.

## 5. Conclusions

We conclude that motor learning objectification during unipedal stance through visual biofeedback of trunk sway smoothness enhances postural control learning, and is useful and reliable for assessing motor learning between days. The results indicate that visual biofeedback of trunk sway smoothness is a good parameter for training postural control. Future studies are needed in clinical and sports contexts to demonstrate the utility of this method.

## Supplementary Materials

The following are available online at https://www.mdpi.com/1424-8220/20/9/2585/s1, Table S1: *P*-value and effect size of comparison between groups and days. Significant differences (*p*-value < 0.05) are bolded.
