# Supplementary material for: Impact of Visual Biofeedback of Trunk Sway Smoothness on Motor Learning during Unipedal Stance
Source: Sensors (Basel). 2020 May 1;20(9):2585. doi: 10.3390/s20092585 (PMC7248825; doi:10.3390/s20092585)
Supplement: Supplementary file 1 [file sensors-20-02585-s001.pdf]

*sensors*

Article

# Impact of Visual Biofeedback of Trunk Sway Smoothness on Motor Learning During Unipedal Stance

**Carlos Cruz-Montecinos [1,2], Antonio Cuesta-Vargas [3,4,5], Cristian Muñoz [1], Dante Flores [1], Joseph Ellsworth [1], Carlos De la Fuente [6,7,8], Joaquín Calatayud [9], Gonzalo Rivera-Lillo [1,10,11], Verónica Soto-Arellano [12], Claudio Tapia [1,13,*] and Xavier García-Massó [14]**

[1] Clinical Biomechanics Laboratory, Department of Physical Therapy, University of Chile, 8380453 Santiago, Chile; carloscruz@uchile.cl
[2] Biomechanics and Kinesiology Laboratory, Hospital San José, 8380419 Santiago, Chile
[3] Department of Physiotherapy, Faculty of Heath Sciences, University of Malaga, 29071 Málaga, Spain; acuesta@uma.es
[4] Institute of Biomedical Research in Malaga. IBIMA, 29010 Málaga, Spain
[5] School of Clinical Science, Faculty of Health Science, Queensland University Technology, Brisbane, QLD 4000, Australia
[6] Carrera de Kinesiología, Departamento de Cs. de la Salud, Facultad de Medicina, Pontificia Universidad Católica, 7820436 Santiago, Chile; delafuentte@gmail.com
[7] Laboratorio LIBFE, Escuela de Kinesiología, Universidad de los Andes, 7620086 Santiago, Chile
[8] Centro de Salud Deportiva, Clínica Santa María, 7520378 Santiago, Chile
[9] Exercise Intervention for Health Research Group (EXINH-RG), Department of Physiotherapy, University of Valencia, 46010 Valencia, Spain; joaquin.calatayud@uv.es
[10] Neuroscience Department, University of Chile, 8380453 Santiago, Chile; gbrivera@uchile.cl
[11] Research and Development Unit, Clínica Los Coihues, 9190025 Santiago, Chile.
[12] Hospital Roberto del Río, 8380418 Santiago, Chile; vsotoarellano@gmail.com
[13] Universidad Tecnológica de Chile INACAP, Escuela Salud, 8340536 Santiago, Chile; ctapiam@inacap.cl
[14] Human Movement Analysis Group (HuMAG), University of Valencia, 46022 Valencia, Spain; xavier.garcia@uv.es
* Correspondence: ctapiam@inacap.cl

Received: 08 April 2020; Accepted: 27 April 2020; Published: 1 May 2020

**Table S1.** P-value and effect size of comparison between groups and days. Significant differences (p-value < 0.05) are bolded.

| **Comparisons between groups** | | Day 1 | Day 2 | Day 3 | Day 4 | Day 5 | Day 6 | Day 7 | Day 8 |
|---|---|---|---|---|---|---|---|---|---|
| | p-value<br>Effect size | 0.201<br>(0.54) | **0.001**<br>(1.52) | **<0.001**<br>(1.74) | **<0.001**<br>(1.96) | **<0.001**<br>(2.36) | **<0.001**<br>(2.76) | **<0.001**<br>(2.90) | **0.004**<br>(1.30) |
| **Comparisons between days** | | | | | | | | | |
| **With first day (No biofeedback)** | p-value<br>Effect size | | 0.999<br>(0.38) | 0.999<br>(0.97) | 0.633<br>(1.41) | 0.334<br>(1.46) | 0.055<br>(1.88) | **0.024**<br>(2.03) | **0.008**<br>(2.13) |
| **With first day (Biofeedback)** | p-value<br>Effect size | | **<0.001**<br>(1.55) | **<0.001**<br>(1.44) | **<0.001**<br>(1.47) | **<0.001**<br>(1.75) | **<0.001**<br>(2.10) | **<0.001**<br>(2.25) | **<0.001**<br>(1.51) |
| | | | | Day 2-3 | Day 3-4 | Day 4-5 | Day 5-6 | Day 6-7 | Day 7-8 |
| **Between consecutives days (No biofeedback)** | p-value<br>Effect size | | | 0.517<br>(1.06) | 0.999<br>(0.67) | 0.999<br>(0.47) | 0.999<br>(0.83) | 0.999<br>(0.60) | 0.999<br>(1.12) |
| **Between consecutives days (Biofeedback)** | p-value<br>Effect size | | | **0.001**<br>(1.19) | **0.004**<br>(1.13) | **0.001**<br>(1.22) | **0.040**<br>(0.84) | **0.048**<br>(0.99) | **<0.001**<br>(1.76) |

© 2020 by the authors. Licensee MDPI, Basel, Switzerland. This article is an open access article distributed under the terms and conditions of the Creative Commons Attribution (CC BY) license (http://creativecommons.org/licenses/by/4.0/).
